# Impaired antiviral activity of interferon alpha against hepatitis C virus 2a in Huh-7 cells with a defective Jak-Stat pathway

**DOI:** 10.1186/1743-422X-7-36

**Published:** 2010-02-11

**Authors:** Sidhartha Hazari, Partha K Chandra, Bret Poat, Sibnarayan Datta, Robert F Garry, Timothy P Foster, Gus Kousoulas, Takaji Wakita, Srikanta Dash

**Affiliations:** 1 Department of Pathology and Laboratory Medicine, Tulane University of Health Sciences Center, 1430 Tulane Ave, New Orleans, LA 70112, USA; 2 Microbiology and Immunology, Tulane University of Health Sciences Center, 1430 Tulane Ave, New Orleans, LA 70112, USA; 3 Division of Biotechnology and Molecular Medicine, School of Veterinary Medicine, Louisiana State University, Baton Rouge, LA 70803, USA; 4 Department of Virology II, National Institute of Infectious Diseases, Tokyo, Japan

## Abstract

**Background:**

The sustained virological response to interferon-alpha (IFN-α) in individuals infected with hepatitis C virus (HCV) genotype 1 is only 50%, but is about 80% in patients infected with genotype 2-6 viruses. The molecular mechanisms explaining the differences in IFN-α responsiveness between HCV 1 and other genotypes have not been elucidated.

**Results:**

Virus and host cellular factors contributing to IFN responsiveness were analyzed using a green fluorescence protein (GFP) based replication system of HCV 2a and Huh-7 cell clones that either possesses or lack a functional Jak-Stat pathway. The GFP gene was inserted into the C-terminal non-structural protein 5A of HCV 2a full-length and sub-genomic clones. Both HCV clones replicated to a high level in Huh-7 cells and could be visualized by either fluorescence microscopy or flow cytometric analysis. Huh-7 cells transfected with the GFP tagged HCV 2a genome produced infectious virus particles and the replication of fluorescence virus particles was demonstrated in naïve Huh-7.5 cells after infection. IFN-α effectively inhibited the replication of full-length as well as sub-genomic HCV 2a clones in Huh-7 cells with a functional Jak-Stat pathway. However, the antiviral effect of IFN-α against HCV 2a virus was not observed in Huh-7 cell clones with a defect in Jak-Stat signaling. HCV infection or replication did not alter IFN-α induced Stat phosphorylation or ISRE promoter-luciferase activity in both the sensitive and resistant Huh-7 cell clones.

**Conclusions:**

The cellular Jak-Stat pathway is critical for a successful IFN-α antiviral response against HCV 2a. HCV infection or replication did not alter signaling by the Jak-Stat pathway. GFP labeled JFH1 2a replicon based stable cell lines with IFN sensitive and IFN resistant phenotypes can be used to develop new strategies to overcome IFN-resistance against hepatitis C.

## Background

Hepatitis C virus (HCV) is the most common blood-borne infection affecting the liver. Chronic HCV infection often leads to the development of liver cirrhosis and cancer [1]. HCV infection often does not present early symptoms and thus can go undetected while significant liver damage sets in over the course of 10-20 years. There are 180 million people currently infected with HCV worldwide [2,3]. The incidence of new HCV infection is increasing each year, creating a significant public health problem. The standard treatment for chronic HCV infection is interferon with ribavirin, but many patients infected with certain viral strains develop resistance to treatment [4,5]. The mechanisms of interferon action and resistance in chronic HCV infection are currently not well understood. Development of efficient HCV cell culture systems for all major HCV strains is required to understand the role of host-virus interaction in the IFN-antiviral response.

HCV, a member of the *Flaviviridae*, is an enveloped virus containing a single-stranded positive sense RNA genome approximately 9600 nucleotides in length [6,7]. The nucleotide sequences of HCV genomes isolated in different parts of world vary considerably and are quite heterogeneous. There are six major genotypes and numerous sub-types of HCV that have been described from all over the world [8-10]. Genotype 1 (subtype 1a and 1b) is the most common in the United States, followed by genotype 2 and 3 [10,3]. Genotype 3 is most common in the Indian subcontinent [8]. Genotype 4 is the most common genotype in Africa and the Middle East [11]. Genotypes 5 and 6 are most common and predominant in South Africa and Southeast Asia [12].

In spite of high sequence variability among different HCV genotypes, the genomic organization of all HCV strains starts with a highly conserved untranslated sequences (called 5' UTR), followed by a large open reading frame, and terminating with 3'-untranslated sequences. The large polyprotein is processed by cellular and viral proteases into structural proteins (core, E1, and E2) and nonstructural proteins (p7, NS2, NS3, NS4A, NS4B, NS5A, and NS5B). The nonstructural proteins NS3 to NS5B are essential for RNA replication and have distinct functions in the HCV life cycle. The 5' and 3' UTR sequences of HCV contain numerous cis-acting signals that are absolutely required for RNA translation and replication as shown by *in vitro *experiments using the cell culture system. Despite the high nucleotide sequence homology of the 5' and 3' UTRs among all genotypes of HCV, the efficiency of RNA genome replication of different HCV strains in the cell culture varies significantly [13]. Some strains of HCV with adaptive mutations replicate efficiently in the cell culture, whereas others do not require any adaptive mutations. The best example is the JFH1 clone of HCV 2a strain that replicates to a higher level in cell culture and generates more infectious virus particles compared to all other full-length infectious clones [14-16]. These observations suggest that HCV genetics and host cellular environments are the two major determinants of the efficacy of HCV replication and its response to antiviral therapy.

Interferon alpha (IFN-α) along with ribavirin has been widely used as a standard treatment option for patients with chronic HCV infection all over the world [3]. However, the sustained virological response to IFN-α in individuals infected with HCV genotype 1 is only 50% as compared with 80% in patients infected with genotype 2 to 6 viruses [17]. Molecular mechanisms explaining why certain genotype viruses respond better to IFN-α than others have not been elucidated. We have shown that IFN-α effectively inhibits the IRES mediated translation of all HCV strains in the cell culture, indicating that differential resistance is not due to IRES sequence heterogeneity [18-20]. To gain an insight into the mechanisms of IFN resistance in the HCV cell culture model, we have developed Huh-7 cell lines in which the HCV 1b Con1 strain is resistant to IFN, after prolonged IFN-α treatment of a low inducer Huh-7 replicon cell line [21,22]. We demonstrated that phosphorylation of Stat1 and Stat2 proteins in the IFN-resistant replicon cell lines is blocked due to reduced phosphorylation of Jak1 and Tyk2 proteins [21,22]. These studies provided direct evidence that a defective Jak-Stat pathway makes HCV replication resistant to interferon treatment in a replicon cell line, and indicated that cellular factors are important for determining the response of HCV to IFN-α treatment. To extend our observations, we have examined the replication and anti-viral response of an IFN-sensitive HCV 2a virus clone in a Huh-7 clone with a defective Jak-Stat pathway. For this purpose, we first developed a chimeric clone between GFP and a highly efficient HCV 2a virus. Insertion of the GFP coding sequences into HCV 2a allowed us to study a high level replication of the virus in Huh-7 cells directly by fluorescence microscopy or flow cytometric analysis. We also determined that replication of HCV 2a can only be inhibited by IFN-α in a dose dependent manner in Huh-7 cells with a functional Jak-Stat pathway. Replication of the full-length and sub-genomic clone of a HCV 2a strain was not inhibited by IFN-α in Huh-7 cell clones with a defective Jak-Stat pathway. Infection with full-length virus or stable replication of sub-genomic HCV RNA did not alter the state of Jak-Stat signaling or interferon sensitivity in these two different Huh-7 clones. We have now developed multiple GFP tagged HCV sub-genomic replicon cell clones in which replication of HCV are totally resistant to IFN-α. We believe that these cell clones can be used to understand the cellular basis of IFN-resistance in a cell culture as well as develop alternative strategies to overcome mechanisms of resistance.

## Materials and methods

### Cell culture

Huh-7.5 cells, obtained from the laboratory of Dr. Charles M. Rice (Center for the Study of Hepatitis C, The Rockefeller University, New York), were cultured at 37°C in Dulbecco's modified Eagle's medium supplemented with 2 mM l-glutamine, nonessential amino acids, 100 U/ml of penicillin, 100 μg/ml of streptomycin and 10% fetal bovine serum, under 5% CO_2 _conditions. Interferon resistant (R-24/1) replicon cells were generated in our laboratory by prolonged treatment of low inducer replicon cell lines (Con-15, Con-17, and Con-24) with IFN-α as described previously [21,22]. A cured Huh-7 cell line with defective Jak-Stat pathway (R-Huh-7) was prepared from IFN-α resistant replicon cell line (R-24/1) after repeated treatment with cyclosporine-A (1 μg/ml) as described previously [22]. Interferon sensitive cured Huh-7 cells (S-Huh-7) were derived from the 5-15 replicon cell line after treatment with IFN-α. Interferon sensitive and interferon resistant phenotypes in the cured S-Huh-7 and R-Huh7 cells were examined by measuring their ability to activate the ISRE-luciferase promoter in the presence of exogenous IFN-α. The expression of functional Jak-Stat signaling proteins in these cells after IFN-α treatment was examined by western blot analysis of phosphorylated Stat1 and Stat2. All the resistant cell lines have defects in the phosphorylation of Stat1 and Stat2 protein, whereas the S-Huh-7 clone showed a high level of phosphorylation of Stat1 and Stat2 proteins within 30 minutes of IFN-α treatment [22]. All Huh-7 cell lines were maintained in Dulbecco's modified Eagle's medium supplemented with 2 mM l-glutamine, nonessential amino acids, 100 U/ml of penicillin, 100 μg/ml of streptomycin and 5% fetal bovine serum.

### Construction of full-length and sub-genomic JFH1 2a chimeric clones

The JFH1 full-length cDNA clone of HCV 2a strain which was isolated from a chronically infected Japanese fulminant hepatitis patient was obtained from Wakita and his coworkers [14]. Chimeric clones between JFH1 and enhanced green fluorescent protein (EGFP) were constructed in our laboratory by the standard overlapping PCR amplification and cloning methods. The coding sequence of GFP was amplified from pEGFP-N1 plasmid and inserted in-frame of the NS5A coding sequence of the JFH1 cDNA clone between 2394 and 2395 amino acids position (between 417 and 418 amino acids of the NS5A protein). The PCR amplification of recombinant DNA and cloning was performed in four different steps. In the first step, the 228 bp (F1) recombinant DNA fragment containing 70 amino acids of NS5A (nts.7339-7546) fusion with the first 6 amino acids of EGFP-N1 was amplified using a sense primer (S/7336-7360/HCV-5'-CCTCCCCCAAGGAGACGCCGGACA-3') and anti-sense primer (AS/7529/HCV- 5'CTCGCCCTTGCTCACCATG GGGGGCATAGAGGAGGC-3'). In the second step, the 719 bp (F2) recombinant DNA fragment containing the total EGFP-N1 open reading frame (ORF) fused with the N- and C-termini of HCV NS5A was amplified using sense and anti-sense overlapping primers (S/7529/GFP- 5'-GCCTCCTCTATGCCCCCCATGGTGAGC AAGGGCGAG-3' and (AS/7547-7564/GFP 5'-TCCAGGCTCCCCCTCGAGCTTGTACA GCTCGTCCAT-3'). In the third step, the recombinant 531 bp DNA fragment (F3) containing last 6 amino acids of EGFP-N1 and 177 amino acids of NS5A (nt. 7547-8077) was amplified by using sense primers (S/7547/HCV- 5'-ATGGACGAGCTGTACAAG CTCGAGGGGGAGCCTGGA-3') and anti-sense primer (AS/8059-8077/HCV-5'-GTCTTCCAGGAGGTCCTTCCACAC-3'). In fourth step, the F1, F2 and F3 PCR fragments were assembled into the 1478 bp DNA fragment through overlapping PCR. In the final step, the recombinant DNA was digested with restriction enzyme *RsrII *and *HpaI*, gel purified and then ligated with pJFH1 clone using the unique *RsrII *and *HpaI *restriction sites present in the NS5A gene. The resulting plasmid was named pJFH1-GFP. The recombinant plasmid was amplified and the construction was confirmed by sequence analysis. A full-length pJFH1-GFP plasmid clone was prepared with a GDD to GND mutation in the NS5B gene to use as a control (pJFH1-GND-GFP) in the replication assay. A full-length pJFH1-GFP plasmid was also prepared with a deletion in the E1-E2 gene (pJFH1-ΔE1E2-GFP) to use as a control in the infectivity assay. To generate a sub genomic GFP replicon clone of HCV 2a, the recombinant plasmid containing the NS5A and EGFP-fusion was excised from full-length pJFH1-GFP plasmid using the *NsiI *and *HpaI *enzyme and re-cloned into the pSGR replicon [23]. This chimeric clone was named pSGR-GFP. As a control, we created a mutant construct pSGR-GND-GFP clone with a point mutation that changes a GDD motif to GND, abolishing the RNA polymerase activity of the NS5B protein. All the recombinant plasmids constructs were confirmed by DNA sequence analysis.

### In-vitro RNA synthesis

Full-length (pJFH1-GFP) and sub-genomic replicon (pSGR-GFP) plasmids were linearized with *XbaI *restriction enzyme and purified by phenol chloroform extraction and precipitated by ethanol. The HCV full length and sub-genomic RNAs were transcribed from *XbaI *linearized plasmid DNA templates using the MEGA-script T7 kit (Ambion, Austin, TX, USA). *In vitro *transcribed RNA was treated with DNase I to eliminate any residual plasmid DNA, extracted with phenol and chloroform, and then precipitated with absolute ethanol. The RNA pellet was re-suspended in nuclease free water and 10 μg aliquots of this RNA were stored at -80°C. The integrity of *in vitro *transcribed RNA was verified by agarose gel electrophoresis.

### RNA transfection

Huh-7.5, S-Huh-7 and R-Huh-7 cells were electroporated with *in vitro *transcribed HCV RNA using a standard protocol described previously [17]. Briefly, cells were harvested using trypsin-EDTA, pelleted by centrifugation and washed in 10 ml of phosphate buffered saline (PBS). The cell pellet was suspended in PBS (10^7 ^cells per ml). Ten micrograms of *in vitro *transcribed RNA was mixed with 400 μl of Huh-7 cell suspension in a cuvette (0.4 cm, Bio-Rad) and subjected to an electric pulse at 960 μF and 270 volts using a gene pulser apparatus (Bio-Rad). Following electroporation, cells were diluted in 10 ml of complete medium and plated in a 100-mm diameter cell culture dish.

### Replication assay

To study replication of full-length HCV-GFP chimeric RNA, the electroporated Huh-7 cells were cultured in a 100-mm plate with regular growth medium. The expression of GFP in the transfected Huh-7 cells was recorded at 0, 24, 48, 72 and 96 hours post-transfection. To study the replication of HCV sub-genomic RNA, stable Huh-7 cells replicating sub-genomic RNA were prepared. Cured Huh-7 cells derived from interferon sensitive (S-Huh-7) and resistant replicon cell lines (R-Huh-7) in our laboratory were used. Huh-7 cells electroporated with sub-genomic RNA were cultured in a growth medium supplemented with 500 μg/ml G-418 drug. These cells were maintained with a regular medium change at every three days for 3-4 weeks until distinct G-418 resistant cell colonies were developed. To make a stable cell line replicating HCV 2a sub-genomic RNA, multiple G-418 resistant cell clones were isolated and cultured in medium supplemented with 1 mg/ml G-418. In these stable cell lines absence of HCV plasmid DNA integration was confirmed by direct PCR followed by Southern blot analysis for the neo gene (sense 5'-ATCGAATTCATCGTGGCTGGCCA-3'; anti-sense 5'-CTAGAATTCGGCGCGAGCCCCTG-3'; probe 5'-GCTTGGTGGTCGAATGGGCAG GTAGCCGGA-3'.

### Infectivity assay

An infectivity assay for HCV was performed using a published protocol [15]. Huh-7.5 cells were transfected with 20 μg of *in vitro *transcribed full-length JFH1-GFP RNA by electroporation method. After 72 h, cells were collected by scraping and then lysed by four rounds of freeze-thaw cycles. The cell lysates were clarified by centrifugation at 3400 rpm for five minutes. The clear supernatant was collected and the titer of HCV in the supernatant was determined by real-time RT-PCR using a primer set targeted to the 5'UTR. A tissue culture infective dose (TCID50) was determined using 10-fold serial dilution of the virus containing supernatant on 2-well Lab-Tek chamber slides (Nalge Nunc International, Rochester, New York). Briefly, Huh-7.5, S-Huh-7 and R-Huh-7 cells were seeded on a 2-well glass chamber slide at a density of 1 × 10^4 ^cells per well. The next day, the culture medium was removed and 1-ml of serial dilutions of culture supernatant containing infectious virus was added to the wells. The cells were incubated overnight at 37°C. On the following day the culture medium was removed, and the cells were washed once using PBS and then incubated in fresh complete medium. After 96 hours post-transfection, the cells were removed, washed in PBS, fixed in 4%-parformaldehyde for 30 minutes and then counter stained with Hoechst dye (H33342, Calbiochem, Darmstadt, Germany) for 15 minutes at 37°C. Cells were examined at 484 nm using a fluorescence microscope (Olympus) for expression of green fluorescence. Cells were then examined at 340 nm for blue nuclear staining. For each area, two sets of pictures were taken. The final image was generated by superimposing blue nuclear fluorescence of Hoechst dye with green fluorescence of GFP using Abode Photoshop software (V 7.0). The numbers of green positive cells in ten different fields were counted and the percentage of green fluorescence positive cells in the culture was determined. The dilution of virus-containing supernatant that showed 50% GFP positive cells 96 hours after infection in the culture (called the TCID50) was determined. The MOI of the infectious culture supernatants was determined by dividing the TCID50 with the cell number used in the infectivity assay.

### Interferon treatment

To study the effect of interferon on the full-length HCV 2a clone, transfected or infected Huh-7 cells were treated with increasing concentrations of IFN-α(Intron A, Schering-Plough, NJ, USA). The antiviral effect of IFN-α against HCV using different Huh-7 clones was confirmed by observing GFP expression under a fluorescence microscope or by flow cytometric analysis, and HCV RNA levels was measured by RPA.

### Ribonuclease protection assay (RPA)

Total RNA was prepared from the cell pellet by the GITC method and subjected to RPA for the detection of genomic positive-strand HCV RNA. For RPA experiments, 25 μg of the total RNA was mixed with a negative-strand RNA probe targeted to the 5'UTR of HCV (1 × 10^6 ^cpm) in a 10 μl hybridization solution, denatured for 3 minutes at 95°C and then hybridized overnight at 50°C. RNase digestion was performed in 200 μl of RNase digestion buffer (10 mM Tris, pH 7.5, 5 mM EDTA and 0.3 M NaCl) containing RNaseA/T1 cocktail at 1:100 dilutions (Ambion Inc., Austin, TX) for an hour at 37°C. Then the sample was treated with 2.5 μl of 25% SDS and 10 μl of proteinase K (20 mg/ml) for 15 minutes. Samples were extracted with phenol and chloroform and then precipitated after addition of 2.5 volumes of absolute ethanol. The pellet was obtained by centrifugation for 30 minutes at 12,000 rpm. The RNA pellet was washed with 70% ethanol, suspended in 8 μl of gel loading buffer, boiled for one minute and separated on a 6% polyacrylamide TBE-Urea gel (Invitrogen, Carlsbad, CA). The gel was dried and exposed to X-ray film (Kodak Biomax-XAR, Rochester, NY). We prepared a plasmid construct called pCR-II (2a), which contained the 79-297 nucleotides of the 5'UTR sequence of the JFH1 clone (pCR-II NT-218) (Invitrogen). This plasmid was linearized with *HindIII *restriction enzyme and a positive strand RNA probe was prepared using T7 RNA polymerase in the presence of 32p labeled CTP. Likewise, this plasmid was linearized with *XbaI *restriction enzyme and Sp6 RNA polymerase was used to prepare a negative strand RNA probe for the detection of a positive strand HCV RNA. The same amounts of the RNA extracts were subjected to RPA for GAPDH mRNA. We used a linearized pTRI-GAPDH-human anti-sense control template to prepare a probe to detect GAPDH mRNA using Sp6 RNA polymerase (Ambion Inc., Austin, TX). The appearance of 218 (HCV 2a) and 317 nts protected fragments indicated the presence of the HCV positive-strand and the GAPDH mRNA, respectively.

### Flow analysis

The percentage of Huh-7 cells expressing GFP after transfection with full-length GFP-RNA transfected cells was analyzed by flow cytometric analysis. Cells were transfected with 10 μg of *in vitro *transcribed RNA in 6-well plates, and harvested by treatment with trypsin-EDTA at 24, 48, 72 and 96 hours post-transfection. The cells were pelleted by centrifugation at 500 rpm in a refrigerated centrifuge. The cell pellet was resuspended in 4% paraformaldehyde for 30 minutes, and washed twice in 10 ml of PBS using centrifugation. After this step, the cell pellet was resuspended in 1 ml of PBS and analyzed by flow cytometer (BD-Biosciences). The percentage of GFP expressing cells in the replicon culture was determined by flow analysis using the identical procedure. Stable replicon cells after interferon treatment were harvested by trypsin-EDTA treatment and analyzed by flow cytometry.

### Real-time RT-PCR

Real time RT-PCR was performed to quantify HCV RNA levels in the infected cell culture using a published protocol [24]. The 243 bp HCV DNA was amplified from the RNA extract by reverse transcription polymerase chain reaction using the outer sense (OS) primer 5'-GCAGAAAGCGCCTAGCCATGGCGT-3' (67-90) and outer anti-sense (OAS) primer 5'-CTCGCAAGCGCCCTATCAGGCAGT-3' (287-310). First the complementary DNA synthesis was performed from positive strand HCV-RNA using an outer anti-sense primer (OAS) targeted to the highly conserved 5'UTR region of HCV in 20 μl volume. Briefly, 2 μgm of total cellular RNA were mixed with 1 μl OAS primer (200 ng/μl), denaturized at 65°C for 10 minutes and annealed at room temperature. Avian myeloblastosis virus (AMV) reverse transcriptase (10 U) (Promega, Madison, WI) was added and incubated at 42°C for 60 minutes in the presence of 50 mmol/L Tris, pH 8.3, 50 mmol/L ethylenediaminetetraacetic acid (EDTA), 500 nmol/L dNTP, 250 nmol/L spermidine, and 40 U RNasin (Promega). The cDNA was stored at -20°C until use. SYBR Green real time PCR amplification was performed in 20 μl of volume containing 10 μl of SYBR Green ER qPCR SuperMix, 1 μl (250 ng/ul) of sense and antisense primer with 4 μl of cDNA and 4 μl of distilled water. All samples were run in triplicate. The amplification was carried out using the standard program recommended by Bio-Rad Laboratory that includes: 50°C for 2 minutes, 95°C for 8 minutes, then additional 50 cycles wherein each cycle consists of a denaturation step at 95°C for 10 seconds, and annealing and extension step at 60°C for 30 seconds. At the end of the amplification cycles, melting temperature analysis was performed by a slow increase in temperature (0.1°C/s) up to 95°C. Amplification, data acquisition, and analysis were performed on CFX96 Real Time instrument (Bio Rad) using CFX manager software (Bio Rad).

## Results

### High-level replication of pJFH1-GFP chimera clone in Huh-7.5 cells

**Figure 1:**
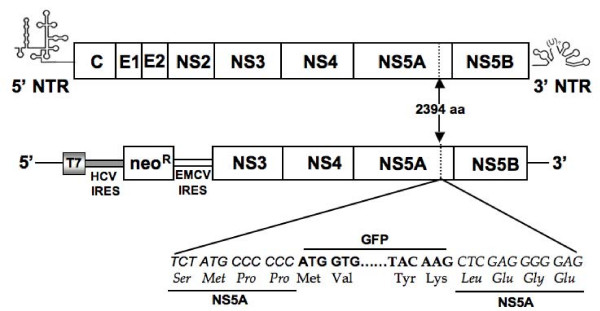
**Structure of HCV full-length and sub-genomic clones used in this project**. The coding sequence of GFP was inserted in frame with the NS5A coding sequence of JFH1 cDNA clone between 2394 and 2395 amino acids position (between 417 and 418 amino acids of NS5A protein). Changes in the nucleotide and amino acid sequences of NS5A gene of HCV-GFP chimera clone are shown. GFP was also inserted at the similar location of NS5A gene (between 417 and 418 amino acids) in the sub-genomic clone, GND mutant clone and E1-E2 deleted mutant clone.

**Figure 2:**
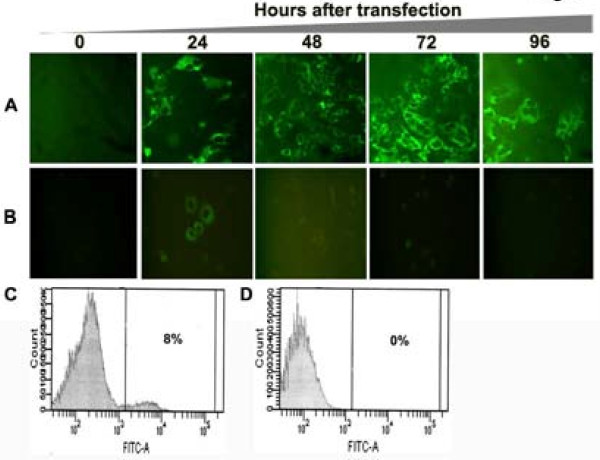
**Replication of JFH1-GFP full-length RNA and JFH1-GND-GFP mutant RNA in Huh-7.5 cells after transfection**. Huh-7.5 cells were electroporated with 10 μg of *in vitro *transcribed RNA prepared either from full-length or GND mutant plasmid. Intracellular expression of GFP in the transfected culture was examined under a fluorescence microscope. (A) Intracellular GFP expression in Huh-7.5 cells transfected with JFH1-GFP RNA at 0, 24, 48, 72 and 96 hours. (B) Intracellular expression of GFP in Huh-7.5 cells transfected with JFH1-GND-GFP mutant RNA at 0, 24, 48, 72 and 96 hours. (C) Intracellular GFP expression measured by flow cytometry in the Huh-7.5 cells transfected with JFH1-GFP RNA after 72 hours. (D): Intracellular GFP expression measured by flow cytometry in the transfected cells of JFH1-GND-GFP mutant RNA after 72 hours.

**Figure 3:**
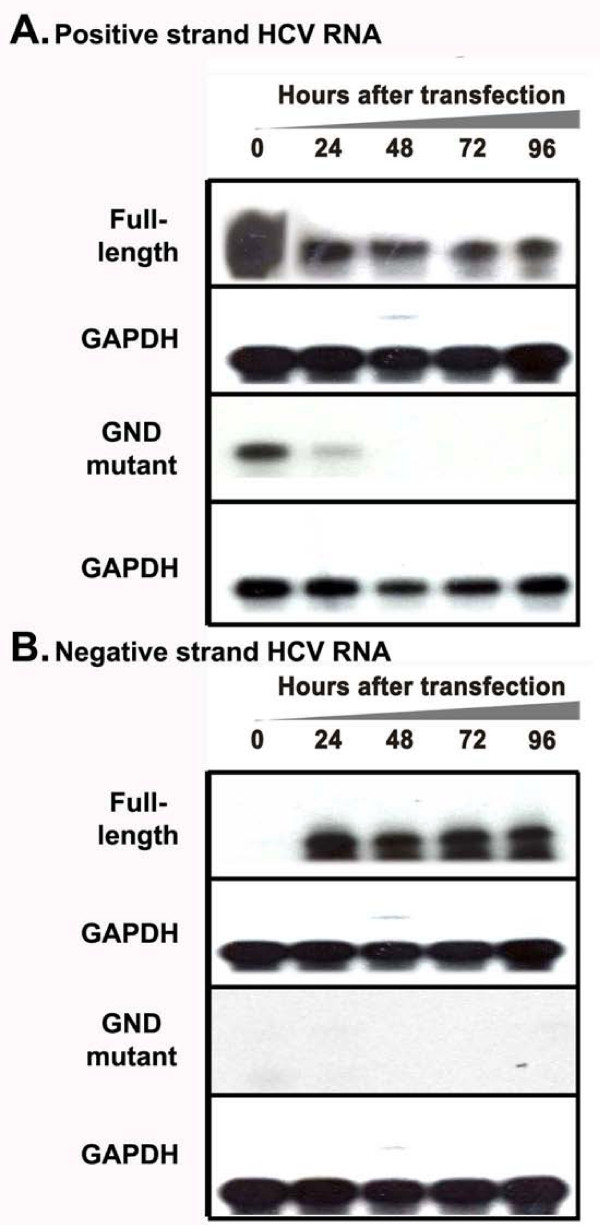
**Detection of positive and negative strand HCV RNA in the transfected Huh 7.5 cells by RPA**. Huh-7.5 cells were transfected with 10 μg of *in vitro *transcribed full-length JFH1-GFP and JFH1-GND-GFP mutant HCV RNA by electroporation. Total RNA was isolated from the RNA transfected cell culture at 0, 24, 48, 72 and 96 hours post-transfection. For the detection of positive strand HCV RNA, total cellular RNA was hybridized with a negative strand RNA probe targeted to the highly conserved 5'UTR region and then RPA experiment was performed. For the detection of negative strand RNA, total cellular RNA was hybridized with a positive sense RNA probe targeted to the 5'UTR region and then RPA was performed. (A) Intracellular HCV positive strand RNA in the Huh-7.5 cells transfected with full-length and mutant JFH1-GFP RNA at 0, 24, 48, 72 and 96 hours post-transfection. GAPDH mRNA levels was used as a loading control. (B) Replicative negative strand HCV-RNA in Huh-7.5 cells transfected with JFH1-GFP and JFH1-GND-GFP mutant RNA. The bottom panel shows the intracellular GAPDH mRNA level indicating that equal amounts of RNA were loaded in each well in the RPA assay.

Replication of the full-length HCV 2a genome is possible due to the availability of the JFH1 cDNA clone. However, the highly sensitive RT-PCR and immunodetection methods are most often needed to detect replication of HCV in the transfected cells. To overcome the technical difficulties associated with the detection of the full-length viral RNA replication, we constructed chimeric clones of the JFH1 clone and GFP so that replication of whole viral genome in the transfected cells could be examined by fluorescence microscopy. Previous reports suggest that heterologous sequences can be inserted into the HCV genome without altering its ability to replicate [25-28]. The coding sequence of GFP was inserted into C-terminus of the NS5A protein of HCV at the 2394 amino acid position. Chimeric clones of GFP and full-length, and a sub-genomic replicon of HCV 2a were prepared (Fig. 1). The N-terminal and C-terminal fusion of HCV NS5A with EGFP protein was confirmed by sequence analysis. To study the replication of full-length virus, *in vitro *transcribed RNA derived from wild type and GND-mutant clone were electroporated into Huh-7.5 cells. The expression of GFP was recorded in a kinetic study. The replication of full-length JFH1-GFP chimera in the transfected Huh-7.5 cells was seen as early as 24 hour post-transfection and the number of GFP positive cells in the culture increased gradually at 48, 72 and 96 hours (Fig 2A). In contrast, replication of the JFH1-GND-GFP mutant RNA in Huh-7.5 cells was not observed at 48, 72 or 96 hours post-transfection, while only a very faint GFP signal was seen in Huh-7.5 cells at 24 hours post-transfection (Fig. 2B). The efficiency of replication of chimeric clones in Huh-7.5 cells after RNA transfection was observed in approximately 8% of cells as examined by flow cytometry (Fig. 2C and 2D). Replication of full-length JFH1-GFP chimera clone was confirmed by examining HCV positive and negative strand RNA levels by RPA assay. The levels of HCV RNA in the full-length transfected cells and GND mutant transfected cells were clearly different (Fig 3A). As expected, the levels of mutant RNA dropped below the input level and remained undetected at 48, 72 and 96 hours post-transfection. The level of HCV positive strand RNA seen in the RPA assay appeared to be higher at an earlier time point in the full-length transfected cells at a later time point. This may be due to an input RNA carryover during the transfection step. There was a good correlation between the amount of HCV RNA and expression of GFP at later time points.

HCV is a positive strand RNA virus that replicates via the synthesis of negative strand RNA. To demonstrate that the replication of transfected RNA resulted in the production of negative strand RNA in the transfected cells, RPA for negative strand HCV RNA was performed in the transfected cells at 0, 24, 48, 72 and 96 hours post-transfection. Negative strand HCV RNA was not detectable at the zero-time point but appeared at 24 hour post-transfection (Fig. 3B). Negative strand RNA was undetectable in Huh-7.5 cells transfected with GND mutant RNA. The presence of negative strand HCV RNA in the full-length transfected cells confirmed active replication of virus in the culture. Based on the results of these experiments we conclude that the chimeric JFH1-GFP clone is replication competent.

**Figure 4:**
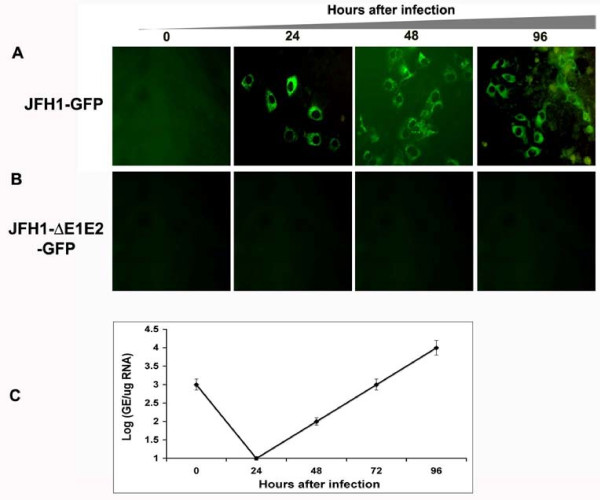
**Infectivity of virus particles produced from Huh-7.5 cells transfected with JFH1-GFP chimeric genome and JFH1-ΔE-E2-GFP deleted mutant clone**. Huh-7.5 cells were transfected with 20 μg of *in vitro *transcribed HCV RNA. After 72 hours, cells along with supernatants were harvested. Four rounds of freezing and thawing using dry ice lysed the cells. Cell free supernatants were collected by centrifugation at 3500 rpm using a tabletop centrifuge. The titer of HCV in the supernatant was determined by real-time RT-PCR. The TCID50 of infectious supernatant was determined by using 10-serial dilution of the virus stock. (A) Intracellular GFP expression in the infected Huh-7.5 cells at 0, 24, 48, 72 and 96 h using MOI of 10 or TCID50 (i.e 10^5 ^virus particle/ml). At different time intervals, cells were taken out from the culture, fixed and GFP examined under a fluorescence microscope. Increased expression of GFP in the infected culture was seen. (B) Intracellular GFP expression in Huh-7.5 cells infected using supernatants of E1-E2 deleted mutant construct. No GFP signal was seen in cells infected using culture supernatants of E1-E2 deleted clone. (C) Real-time RT-PCR was used to quantify the HCV RNA level in the infected cells using a primer targeted to the HCV 5'UTR region. HCV RNA titer in the infected cultures was increased with time suggesting that replication of HCV genome in the infected culture.

To examine infectious virus particle production from cells transfected with JFH1-GFP chimera RNA, an infectivity assay was performed. Culture supernatants were collected from transfected cells, clarified by centrifugation and inoculated to Huh-7.5 cells. The infectivity of HCV was confirmed by direct examination of infected cells under a fluorescence microscope and HCV RNA levels were measured by real-time RT-PCR assay. Infectivity of culture supernatants from cells transfected with full-length and E1-E2 deleted mutant clone was determined by measuring intracellular GFP expression. There was an increase in the number of GFP positive cells after 24, 48 and 72 hours suggesting the replication of HCV RNA after natural infection (Fig. 4A). No GFP expression was observed in Huh-7.5 cells infected with supernatants derived from cells transfected with E1-E2 deleted mutant HCV RNA (Fig. 4B). To confirm that the expression of HCV in the infected cells is associated with the increase in viral RNA, the titer of HCV positive strand RNA was measured using a real-time RT-PCR. The level of HCV RNA in the infected cell cultures was increased from 24 to 72 hours suggesting the replication of HCV-RNA genome in the infected culture (Fig. 4C). Thus, JFH1-GFP-tagged HCV RNA genome is able to replicate in Huh-7.5 cells after transfection and generates an infectious virus.

### High-level replication of GFP labeled sub-genomic RNA of HCV 2a clone

**Figure 5:**
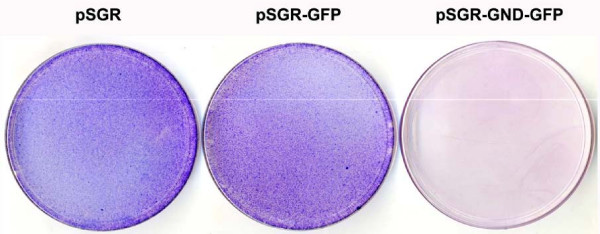
**Replication of unmodified sub-genomic HCV 2a RNA clone and GFP labeled sub-genomic HCV 2a chimera in Huh-7 cells**. Huh-7 cells were transfected with 10 μg of in vitro transcribed RNA by the electroporation method and then cultured in the medium containing G-418 (500 μg/ml). After 4-weeks, G-418 resistant cell colonies were stained with Giemsa Stain (Sigma Chemical). Both the unmodified (pSGR) and GFP tagged sub-genomic clone (pSGR-GFP) replicated at equal efficiencies based on the development of number of G-418 resistant Huh7 cell colonies. No G-418 resistant colonies were present in Huh-7 cells with GND mutant sub-genomic RNA with GFP (pSGR-GND-GFP).

**Figure 6:**
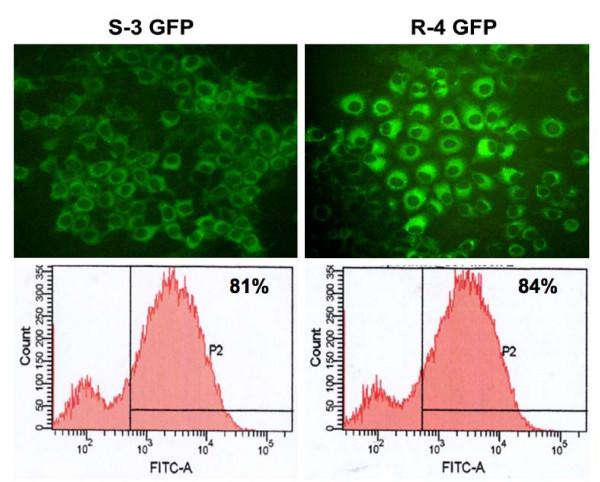
**Preparation of stable replicon cell line replicating HCV 2a sub-genomic RNA**. Interferon sensitive (S3-GFP) and interferon resistant (R4-GFP) Huh-7 cells were transfected with pSGR-GFP replicon RNA and then selected with G-418 (500 μg/ml). Single G-418 resistant cell clones were picked and stable cell lines were generated. (A) Intracellular GFP expression in S3-GFP and R4-GFP stable replicon cell lines. (B) Quantification of GFP expression in these IFN-sensitive (S3-GFP) and resistant (R4-GFP) cells was performed flow analysis. Approximately 81% of S3-GFP and 84% of R4-GFP cells in the culture showed intracellular GFP expression. High-level expression of HCV-GFP was seen in both cell lines suggesting that both the sensitive and resistant Huh-7 clones support high level HCV replication.

Since the JFH1 2a clone replicates to a high level in a cell culture without adaptive mutations, we attempted to develop stable replication competent Huh-7 cells containing GFP labeled sub-genomic HCV RNA. The availability of these cell lines allowed us to reliably quantify the antiviral effect of IFN-α. A chimeric clone combining GFP and sub-genomic clone was prepared. As a control, GND mutant (pSGR-GND-GFP) for the replicon clone was also prepared. The full-cycle replication of pSGR-GFP RNA and unmodified pSGR-RNA in Huh-7 cells were compared for their ability to form cell colonies when cultured in the presence of a medium containing G-418 (500 μg/ml). In this assay, the cells supporting HCV RNA replication survived G-418 drug selection and formed cell colonies. No noticeable differences in the efficiency of replication of the sub-genomic RNA with or without GFP insertion in the NS5A region were observed based on the number of G-418 resistant cell colonies that appeared on the plate (Fig. 5). No colonies developed in the culture transfected with the GND mutant sub-genomic HCV RNA. Individual cell colonies were picked and stable Huh-7 cell lines supporting replication of HCV-GFP sub-genomic RNA were developed. The absence of stable DNA integration in these cell lines was confirmed by direct PCR analysis for neo gene followed by southern blot analysis. High levels of GFP expression due to replication of sub-genomic HCV 2a clone was seen in sensitive and resistant Huh-7 clones (Fig. 6A). The expression of HCV-GFP chimera protein was seen exclusively in the cytoplasm in the majority of Huh-7 cells in the culture. These cell lines have maintained stable GFP expression over more than one year when cultured in a growth medium supplemented with G-418 (500 μg/ml). Two types of stable replicon cell lines were prepared using Huh-7 cells with or without functional Jak-Stat pathway. Stable HCV-GFP replicon cell lines prepared using IFN sensitive (S-Huh-7) cells were named as S3-GFP and S10-GFP replicons. Replicon cell lines, also prepared using IFN resistant Huh-7 cell lines (R-Huh-7), were named as R4-GFP and R8-GFP replicons. The level of GFP expression in the IFN sensitive and resistant replicon Huh-7 cell lines was quantitatively determined by flow analysis. The results of these experiments suggest that more than 80% of replicon cells express GFP (Fig. 6B).

### Antiviral activity of IFN-α against full-length HCV 2a is blocked in Huh-7 cell clone (R-Huh 7) with a defective Jak-Stat pathway

**Figure 7:**
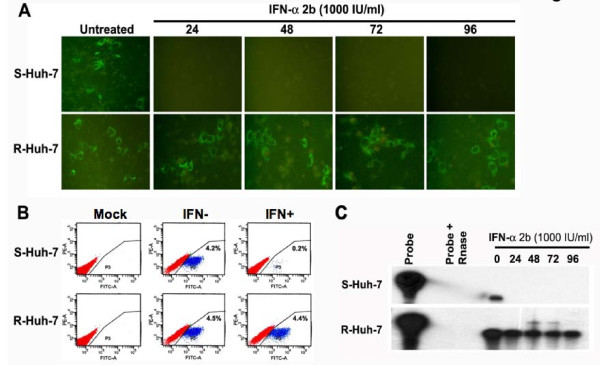
**Antiviral effect IFN-α on the replication of JFH1-GFP RNA using S-Huh-7 and R-Huh-7 cells in culture**. (A) S-Huh-7 and R-Huh-7 cells were transfected with full-length JFH1-GFP RNA and next day the cultures were treated with IFN-α (1000 IU/ml). Intracellular GFP expression was measured after 24, 48, 72 and 96 hours of post-transfection. Upper panel shows the intracellular GFP expression after IFN-α treatment in S-Huh-7 cells. Bottom panel shows the intracellular GFP expression in R-Huh-7 cells after IFN-α treatment (B) Quantification of antiviral effect of IFN-α (1000 IU/ml) between these two cell lines by flow analysis after 72 hours of post-transfection. In the S-Huh-7 cell line the number of GFP positive cells were decreased from 4.2% to 0.2% after IFN-α treatment. There is no decrease in the number of GFP positive cells after IFN-α treatment in the transfected R-Huh-7 cells. The upper and lower left panel shows the untransfected cells as mock. (C) Intracellular HCV mRNA levels in the IFN treated cells measured by RPA. The experiments were carried out same way as described in the panel A; except that the RPA analysis was performed using the total RNA isolated from the transfected cells at different time points. Upper panel shows the HCV RNA level in the transfected S-Huh-7 cells after IFN-α treatment. Bottom panel shows the HCV RNA levels in the transfected R-Huh-7 cells after IFN-α treatment.

**Figure 8:**
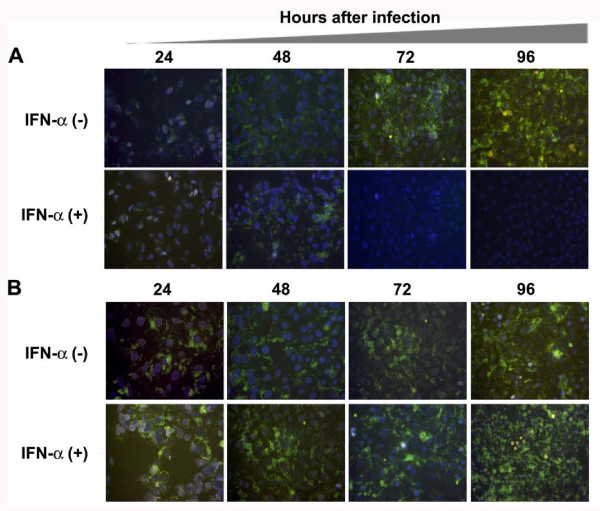
**Antiviral effect of IFN-α against HCV replication in the infected Huh-7 cells in culture**. S-Huh-7 and R-Huh-7 cells were seeded at density of 1 × 10^4 ^cell per well in a chamber slide. The next day cultures were infected with infectious HCV GFP virus at the TCID50 (1 × 10^5 ^virus particles/ml) or MOI of 10 using the standard protocol described in the material and method section. After 24 hours cultures were treated with IFN-α (1000 IU/ml). (A) Intracellular GFP expression in the infected S-Huh-7 cells in culture at 24, 48, 72 and 96 hours of post-infection in the presence and absence of IFN-α. The results show that HCV RNA replication in the infected cells can be inhibited over time by treatment with alpha interferon in S-Huh-7 cells. (B) Intracellular GFP expression in the infected R-Huh-7 cells in culture at 24, 48, 72 and 96 hours of post-infection in the presence and absence of IFN-α. The results suggest that HCV RNA replication remain resistant to IFN-α treatment at all time points in infected R-Huh-7 cells with a defective Jak-Stat pathway.

The development of JFH1-GFP chimera using the HCV 2a clone allowed us to quantify the antiviral properties of IFN-α in Huh-7 cells. One important predictive factor associated with IFN response is the viral genotype. It has been reported by a number of investigators that the sustained virological response in patients infected with HCV genotype 2 is much higher than in patients infected with genotype 1 virus. We conducted experiments to determine whether the replication of an HCV 2a strain could be inhibited in liver cells (R-Huh-7) having a defective Jak-Stat pathway. Both S-Huh-7 and R-Huh-7 cells were transfected individually with full-length JFH1-GFP RNA and then treated with an increasing concentration of IFN-α. We first determined that both S-Huh-7 and R-Huh-7 cells developed in our laboratory supported HCV 2a replication and infection. The ability of IFN-α to inhibit full-length HCV 2a replication in these two different Huh-7 clones was examined in a kinetic study at 24 to 96 hours. Results shown in the upper panel of Fig. 7A suggest that GFP expression can be efficiently inhibited in S-Huh-7 cell clones. There was no reduction in GFP expression in the R-Huh-7 cell clones with a defective Jak-Stat pathway at all time points (lower panel of Fig. 7A). The antiviral effect of IFN-α against HCV 2a in these two cell clones (S-Huh-7 and R-Huh-7) was also quantified by flow cytometric analysis. We found a time dependent effect of IFN-α on HCV 2a replication in S-Huh-7 cells and the number of GFP positive cells was decreased from 4.2% to 0.2% as compared to resistant Huh-7 cell line (Fig. 7B). To verify that the inhibition of GFP is also associated with the reduction of viral RNA in the interferon treated cells, RNA extracts were assayed for HCV RNA by RPA assay using a probe targeted to the 5' UTR region of HCV genome. We found that interferon treatment decreased HCV RNA levels in S-Huh-7 clones and the levels of HCV RNA remained unchanged after interferon treatment in the resistant clone. (Fig. 7C). The ability of IFN-α to stop viral RNA replication in the infected cells was also examined using these two Huh-7 cell clones. IFN-α treatment efficiently inhibited HCV replication as measured by GFP expression in S-Huh-7 cells within 24 hours (Fig 8A). However, antiviral activity of IFN-α against the full-length HCV 2a replication was prevented in R-Huh-7 cells with the defective Jak-Stat pathway (Fig. 8B). The results of these experiments indicate that antiviral activity of IFN-α to inhibit replication of full-length HCV 2a clone was prevented in R-Huh-7 clone with defective Jak-Stat pathway.

### Antiviral activity of IFN-α is impaired against HCV 2a sub-genomic clone in Huh-7 cell clone with a defective Jak-Stat pathway

**Figure 9:**
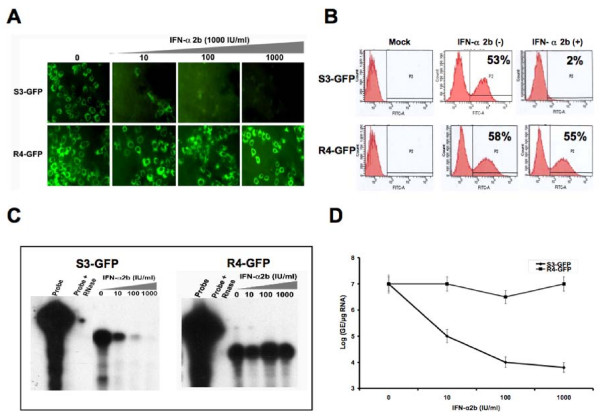
**Antiviral effect of IFN-α using stable cell lines replicating GFP labeled sub-genomic RNA of HCV 2a**. S3-GFP and R4-GFP were treated with IFN-α from 10 to 1000 IU/ml. (A) Upper panel shows the antiviral effect of IFN-α against HCV 2a sub-genomic RNA replication in S3-GFP cells. There was a dose dependent decrease in the GFP expression in the S3-GFP cells with increasing concentration of IFN-α. Lower panel shows no effect of GFP expression in R4-GFP cells. (B) The GFP fluorescence was quantitatively measured by flow cytometry analysis after IFN-α treatment (1000 IU/ml) for 72 hours using S3-GFP and R4-GFP cells. A significant reduction in the number of GFP positive S3-GFP cells (53% to 2%) was seen after IFN treatment. The number of GFP-positive cells did not decrease when treated with interferon (58% to 55%) using R4-GFP cells. (C) RPA shows the intracellular HCV RNA level in the S3-GFP and R4-GFP after IFN-α treatment. Left panel shows HCV RNA levels in S3-GFP after IFN-α treatment at 72 hours. Right panel shows the HCV RNA level in R4-GFP after IFN-α treatment at 72 hours. There is a gradual reduction of HCV mRNA level in the S3-GFP replicon cells compared to the R4-GFP cells. (D) Shows the quantification of HCV RNA levels in the S3-GFP and R4-GFP after IFN-α treatment at 72 hours by real-time RT-PCR. In both the cells the HCV RNA level was detected up to a level of titer of log 7 GE/μg of total RNA. The HCV RNA levels reduced significantly after IFN-α treatment at 1000 IU/ml in the S3-GFP replicon cells. There was no decrease in the HCV RNA level in R4-GFP cells after IFN-α treatment.

The role of the Jak-Stat pathway in the IFN-α response to HCV 2a was also studied using an IFN sensitive (S3-GFP) and IFN resistant (R4-GFP) stable Huh-7 cell line that replicates sub-genomic RNA. Replication of HCV 2a sub-genomic RNA in the S3-GFP after IFN-α treatment was studied by measuring the intracellular GFP expression directly under a fluorescence microscope. It was found that GFP expression in the stable cell line (S3-GFP) diminished over time (Fig 9A). Where as no reduction of the HCV-GFP signal in R4-GFP replicon was observed even when treated with a similar concentration of IFN-α for an extended period. To quantify the IFN antiviral effect intracellular GFP expression was analyzed by flow analysis. The GFP peak disappeared after IFN treatment only in the S3-GFP replicon cell line (53% to 2%). The percentage of GFP positive cells did not decrease (58% to 55%) when similar experiments were carried out using R4-GFP cells (Fig. 9B). To correlate the results of GFP expression, intracellular HCV RNA after IFN-α treatment was also measured by RPA. The results of RPA assays demonstrate that HCV RNA replication is not inhibited by IFN-α treatment in the R4-GFP replicon cell line (Fig. 9C). The level of HCV RNA was also quantified by real-time PCR in these two cell lines after IFN treatment. IFN-α treatment inhibited the HCV RNA level in a dose dependent manner in S3-GFP but the HCV RNA level remained the same in the R4-GFP replicon. There was a significant difference in the level of HCV RNA between the IFN sensitive replicon and resistant replicon after IFN treatment measured by real-time PCR (Fig. 9D). These results suggest that replication of HCV 2a full-length as well as sub-genomic RNA can not be inhibited by IFN-α in R-Huh-7 cells with a defective Jak-Stat pathway.

### HCV infection and replication did not alter the state of Jak-Stat pathway in S-Huh-7 and R-Huh-7 cell clones

**Figure 10:**
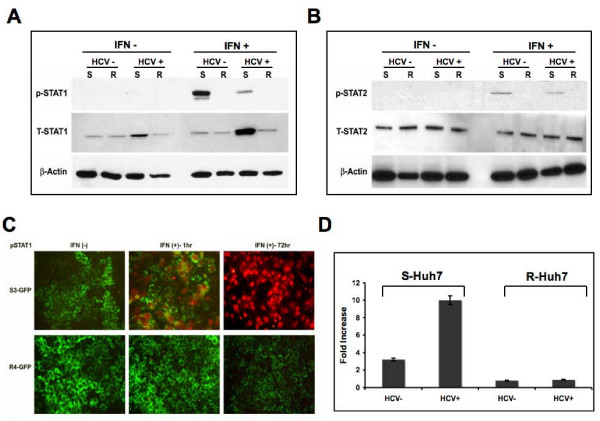
**IFN-α induced Jak-Stat signaling in both S-Huh-7 and R-Huh-7 cells after HCV 2a infection**. (A) Both S-Huh-7 and R-Huh-7 cells were infected with JFH1-GFP chimera virus for 96 hours. The phosphorylation of Stat1 protein in the infected cultures after 30 minutes of IFN-α treatment (1000 IU/ml) was determined by western blot analysis. Equal amounts of proteins were used in the blot to assay for total Stat1 and beta-actin levels. (B) IFN-α induced pStat2 protein in S-Huh-7 and R-Huh-7 cells with or without HCV 2a infection. The experiment was carried out as described in panel A. Equal amounts of proteins were used in the blot to assay for total Stat2 and beta-actin levels. (C) Demonstrates IFN-α induced nuclear localization of pStat1 in the S3-GFP and R4-GFP stable replicon cells at 24 and 72 hours by immunofluorescence microscopy using anti-mouse pStat1 (1:1000 dilution) and Alexaflour labeled anti-mouse secondary antibody (1:2000 dilution). The pStat1 protein was detected in the nucleus of S3-GFP replicon cells only at 24 and 72 hours after IFN-α treatment. The pStat1 translocation is associated with negative GFP expression after 72 hours of IFN-α treatment only in S3-GFP cells. (D) IFN-α induced ISRE-luciferase activity in S-Huh-7 and R-Huh-7 cells after HCV 2a infection. Both S-Huh-7 and R-Huh-7 cells were were infected with full-length HCV. After 72 hours, infected cells were transfected with μgm of pISRE-luciferase plasmid. After 24 hours, ISRE-luciferase activity in the cell lysates were measured in the presence and absence of IFN-α(1 IU/ml) treatment for 24 hours. The values were calculated as fold increase with respect to untreated cells.

Experiments were carried out to examine whether infection or replication of HCV in both S-Huh-7 and R-Huh-7 cells could have any impact on the IFN-α induced Jak-Stat signaling. The levels of pStat1 and pStat2 proteins in the lysates of S-Huh-7 and R-Huh-7 cells after 96 hours of HCV infection were examined by western blot analysis. Results shown in Fig. 10A and 10B clearly show that IFN-α treatment induced pStat1 and pStat2 protein in the infected as well uninfected S-Huh-7 only. However, pStat1 or pStat2 protein was not detected in the infected R-Huh-7 cells even after interferon treatment. These results were confirmed by a co-localization of pStat1 protein in the GFP labeled replicon cells after IFN-treatment. We show that pStat1 is induced only in the sensitive replicon (S3-GFP) and localizes to the nucleus. The nuclear translocation of pStat1 is correlated with a decrease in GFP expression after IFN-α at 72 hours in the S-Huh-7 cells only (Fig. 10C). The pStat1 protein was undetectable in R4-GFP cells after IFN-α treatment. To examine if the effect of HCV infection or replication in both S-Huh-7 and R-Huh-7 could alter the overall Jak-Stat signaling, the ISRE-luciferase promoter activity was examined by a transient transfection assay. Interferon induced activity of ISRE-luciferase did not change significantly in R-Huh-7 cells after HCV infection (Fig. 10D).

## Discussion

The JFH1 full-length cDNA clone of HCV 2a strain was isolated from a chronically infected Japanese patient by Wakita and his coworkers [14]. JFH1 derived clones replicate at a greater efficiency than all other HCV strains, making it the system of choice for biochemical studies that address HCV replication mechanisms and virus host interactions. The replication of full-length virus in cell culture is assessed by the detection of viral RNA by using a highly sensitive RT-PCR method. Viral proteins were detected by western blot analysis, ELISA or immunocytochemistry. These methods are highly specific and accurately determine the replication kinetics but are complex and time consuming. To overcome these difficulties, we prepared a chimeric clone of JFH1 by inserting the coding sequence of EGFP-N1 in the NS5A coding sequences. We noticed that a high-level expression of this JFH1-GFP chimera was seen in Huh-7.5 cells 24 h after transfection. The expression of GFP in the transfected cells is an indication of active replication of the HCV genome since no GFP expression was detected in cells transfected with a GND mutant RNA. Replication of JFH1-GFP RNA in the transfected cell is supported by the results of detection of positive and negative strand RNA. We also showed that the transfected cells produced infectious virus particles. The infection can be transferred to naïve Huh-7 cells in a culture. The expression of GFP protein and viral RNA increased over time in the infected culture suggest that the replication of HCV occurred over time after natural infection. We also prepared a JFH1 sub-genomic clone with GFP as a fusion protein. Multiple stable replicon cell lines containing the GFP chimeric clone and neomycin selection marker were prepared in Huh-7 cells. Replication of sub-genomic clone of HCV 2a in the Huh-7 cells was stable. High-level expression and replication of HCV sub-genomic RNA was observed in the cells for over one year, and can be assayed by flow analysis. Stable cell lines replicating HCV sub-genomic RNA were prepared using IFN-sensitive (S-Huh7) and resistant Huh-7 cells (R-Huh7). We now clearly showed that replication of HCV-GFP chimera cannot be inhibited by IFN-α in Huh-7 cells with defective Jak-Stat pathway.

The availability of a full-length GFP clone and stable replicon cell lines have allowed us to examine the antiviral mechanisms of IFN-α against HCV 2a strain in cell culture. There are reports suggesting that the effectiveness of the IFN response depends on the viral genotype. We performed a study to examine differences in the level of IFN response of HCV using the HCV 2a replication system. Previously, we have demonstrated that both the HCV 1a and HCV 1b strain can be efficiently inhibited by IFN-α within 72 h in a concentration dependent manner [18,19]. In this study we provide evidence suggesting that interferon alpha treatment inhibited HCV RNA replication of full-length as well as replication of HCV sub-genomic RNA in a dose dependent manner. These results are also consistent with a previous report suggesting that IFN inhibits replication of HCV 2a and HCV 1b strain in a dose dependent manner [29]. The role of virus and the Jak-Stat pathway of host cell in the IFN response using HCV 2a cell culture system were examined. We showed here that IFN-α treatment induced phosphorylation of Stat1 and Stat2 proteins in the infected S-Huh-7 cells and successfully inhibited HCV RNA replication. However, we could not detect phosphorylated Stat1 or Stat2 protein in the HCV infected R-Huh-7 cells after IFN-α treatment. The IFN-α induced Jak-Stat mediated ISRE-luciferase activity of R-Huh-7 cells did not change significantly with or without HCV infection. We showed here that IFN-α is not able to inhibit the replication of full-length as well as sub-genomic HCV 2a virus in R-Huh-7 cell clone suggesting a dominant role of cellular Jak-Stat pathway in the response to the interferon treatment [22].

The overall sustained virological response of patients infected with HCV genotype 2 and 3 is about 80% as compared to only 50% in the case of chronic HCV patients that are infected with HCV genotype 1 strain [3]. The mechanisms that determine the response at the genotype level are not clear. There has been a report suggesting that the IFN treatment response alters two phases of viral replication kinetics [30]. The first phase is the dose dependent reduction of HCV RNA levels in the liver within the first 24 hours after treatment. The second phase of IFN-induced decline of HCV RNA occurs over weeks to months. The first phase viral decay may be due to the direct action of interferon on HCV production and the second phase may be due to death of infected cells. In our analysis, we have found that there is no difference in the efficacy of IFN upon replication of HCV 2a and HCV genotype 1b viruses. It will be important to determine if there are differences in death of hepatocytes when they are infected with HCV genotype 1 and HCV 2a virus. Our study provides evidence suggesting that cells with defective Jak-Stat pathway of IFN-signaling can prevent the antiviral response after IFN-α treatment. This conclusion supports results from previous studies using HCV cell cultures [22,31] as well as by a recent multicenter study using clinical samples from HALT-C trial suggesting that response to IFN therapy is dependent upon the host genetic polymorphisms of Tyk2 in the liver cells [32]. In summary, results of this investigation support the importance of host cell factors in the mechanisms of IFN-resistance in chronic HCV infection. The development of IFN-sensitive and IFN-resistant GFP tagged HCV 2a replicon cell lines will allow us to further understand the mechanisms of resistance against HCV in tissue culture. In particular, GFP labeled IFN-resistant replicon cells should be very useful to develop alternative antiviral strategies to overcome IFN resistance against HCV.

## Competing interests

The authors declare that they have no competing interests.

## Authors' contributions

SH performed most of the biochemical experiments, prepared the chimeric constructs, cell lines, and participated in the design of the study. PKC contributed in the full-length infectivity assay. BP prepared RPA probe for negative strand detection and SND helped in western blot experiments. TPF and GK provided us the temporary laboratory space to recover the cell lines at the time of hurricane Katrina. TW provided the initial JFH1 constructs. RFG helped in editing the manuscript. SD overall supervised, helped to design the study and wrote the manuscript. All authors read and approved the final manuscript.
